# Supplementary material for: Host heparan sulfate promotes ACE2 super-cluster assembly and enhances SARS-CoV-2-associated syncytium formation
Source: Nat Commun. 2023 Sep 18;14:5777. doi: 10.1038/s41467-023-41453-w (PMC10507024; doi:10.1038/s41467-023-41453-w)
Supplement: Supplementary file 1 — Supplementary Information [file 41467_2023_41453_MOESM1_ESM.pdf]

Supplemental Information

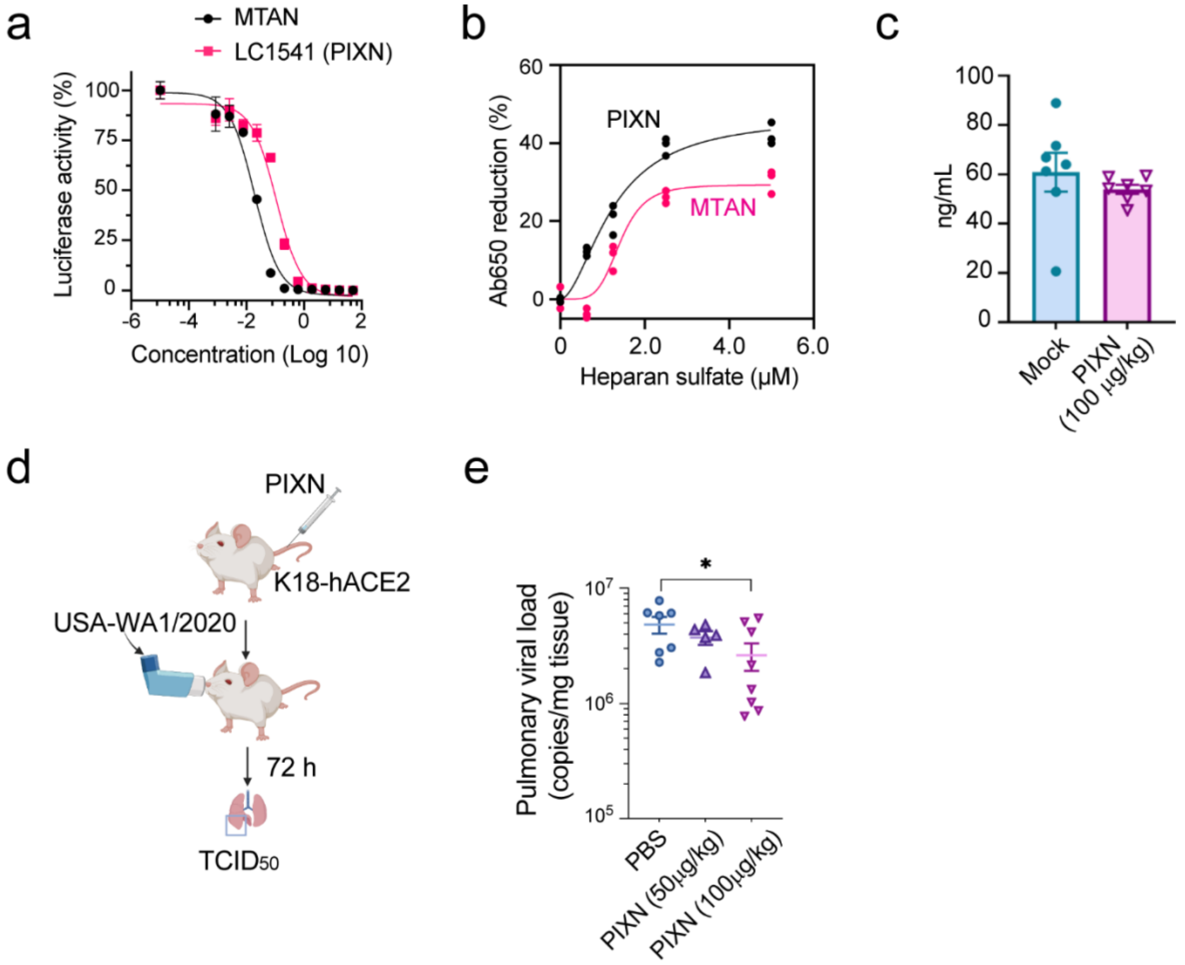

Supplementary Fig. 1: PIXN blocks endocytosis-mediated entry of SARS-CoV-2.

a PIXN (LC1541) and MTAN inhibit the entry of luciferase-expressing pseudoviral particles coated with the spike of the SARS-CoV-2 Omicron variant. Error bars indicate means ± SD, n=3 biological repeats.

b Heparan sulfate binds to PIXN and MTAN. The indicated drugs (50 μM) were incubated with purified heparan sulfate at the indicated concentrations. The change of absorbance at 650 nm was recorded. The fitted curves represent the average of three biological repeats.

c Naïve Balb/c mice were injected via i.p. with 100 µl/mouse of vehicle (Mock) or PIXN (100 µg/kg) and were tail-bled 3 days later. Serum aspartate aminotransferase (AST) was measured using a mouse AST ELISA kit (MyBioSource). Error bars indicate means ± SD, n=7 mice.

d The experimental scheme for *in vivo* testing of PIXN in K18-hACE2 transgenic (Tg) mice. Created by BioRender.com.

e PIXN reduces SARS-CoV-2 infection in K18-hACE2 mice. Mice were pretreated with PIXN at the indicated doses followed by intranasal infection with live USA-WA1/2020. Viral load in the lung was determined 72 h post infection by qRT-PCR. Error bars indicate means ± s.e.m. *, p=0.03 by two-sided unpaired Mann-Whitney test. n=7, 5, 8 mice for the three groups as indicated.

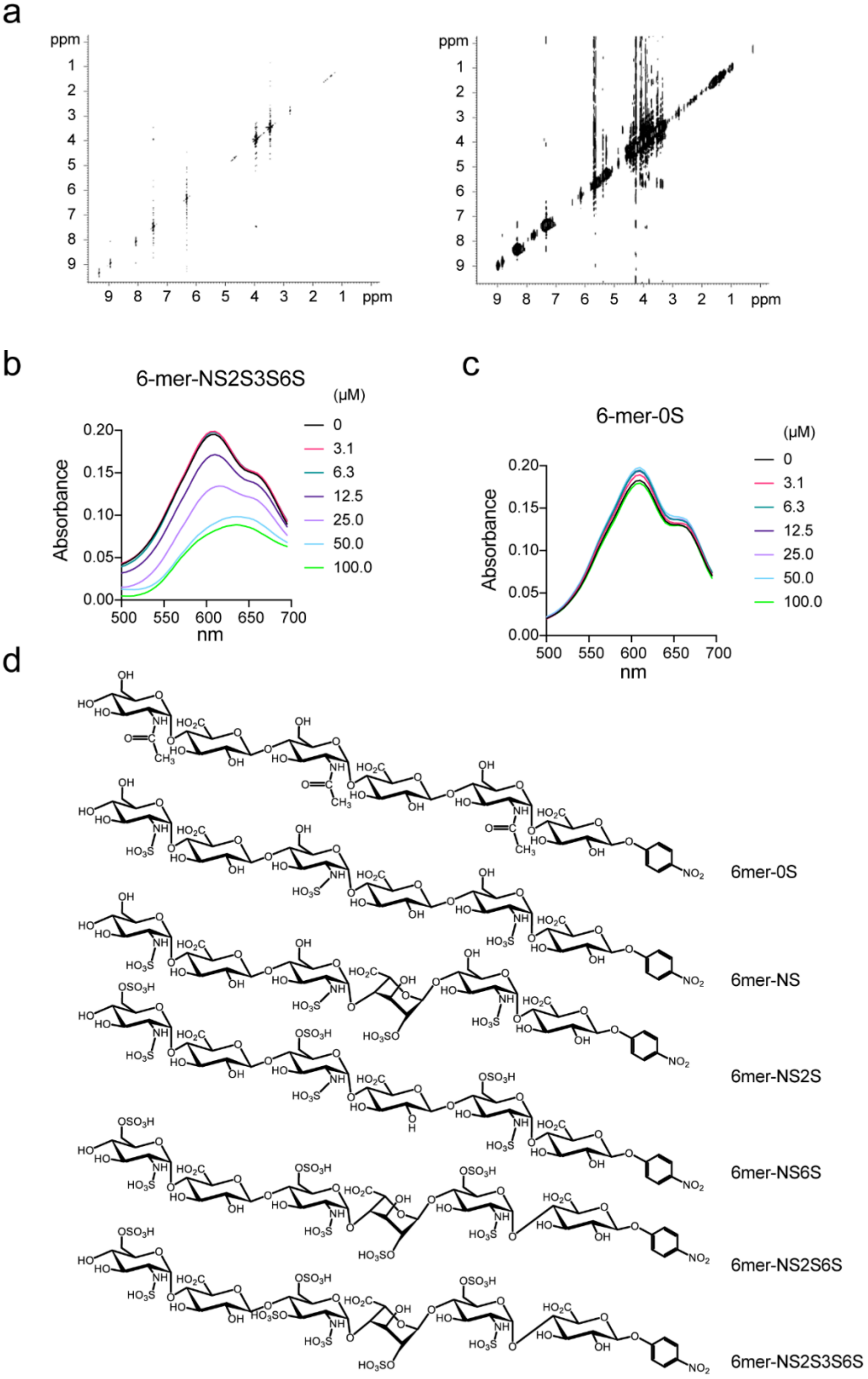
a
ppm
1
2
3
4
5
6
7
8
9
9 8 7 6 5 4 3 2 1 ppm
b
6-mer-NS2S3S6S
(μM)
0
3.1
6.3
12.5
25.0
50.0
100.0
Absorbance
0.00
0.05
0.10
0.15
0.20
500 550 600 650 700
nm
c
6-mer-0S
(μM)
0
3.1
6.3
12.5
25.0
50.0
100.0
Absorbance
0.00
0.05
0.10
0.15
0.20
500 550 600 650 700
nm
d
6mer-0S
6mer-NS
6mer-NS2S
6mer-NS6S
6mer-NS2S6S
6mer-NS2S3S6S

Supplementary Fig. 2: Pixantrone (PIXN) and Mitoxantrone (MTAN) bind HS 6-mer NS2S3S6S in a sulfate dependent manner.

a NOE spectra of free PIXN (Left) and PIXN + HS 6-mer NS2S3S6S (Right). Both spectra were acquired using 120 msec mixing time.

b MTAN (50 μM) was incubated with HS 6-mer NS2S3S6S at the indicated concentrations (μM). The absorbance spectra of the reactions were measured.

c As in **b**, except that a HS 6-mer with zero sulfate was used.

d The structures of HS 6-mers used in the study.

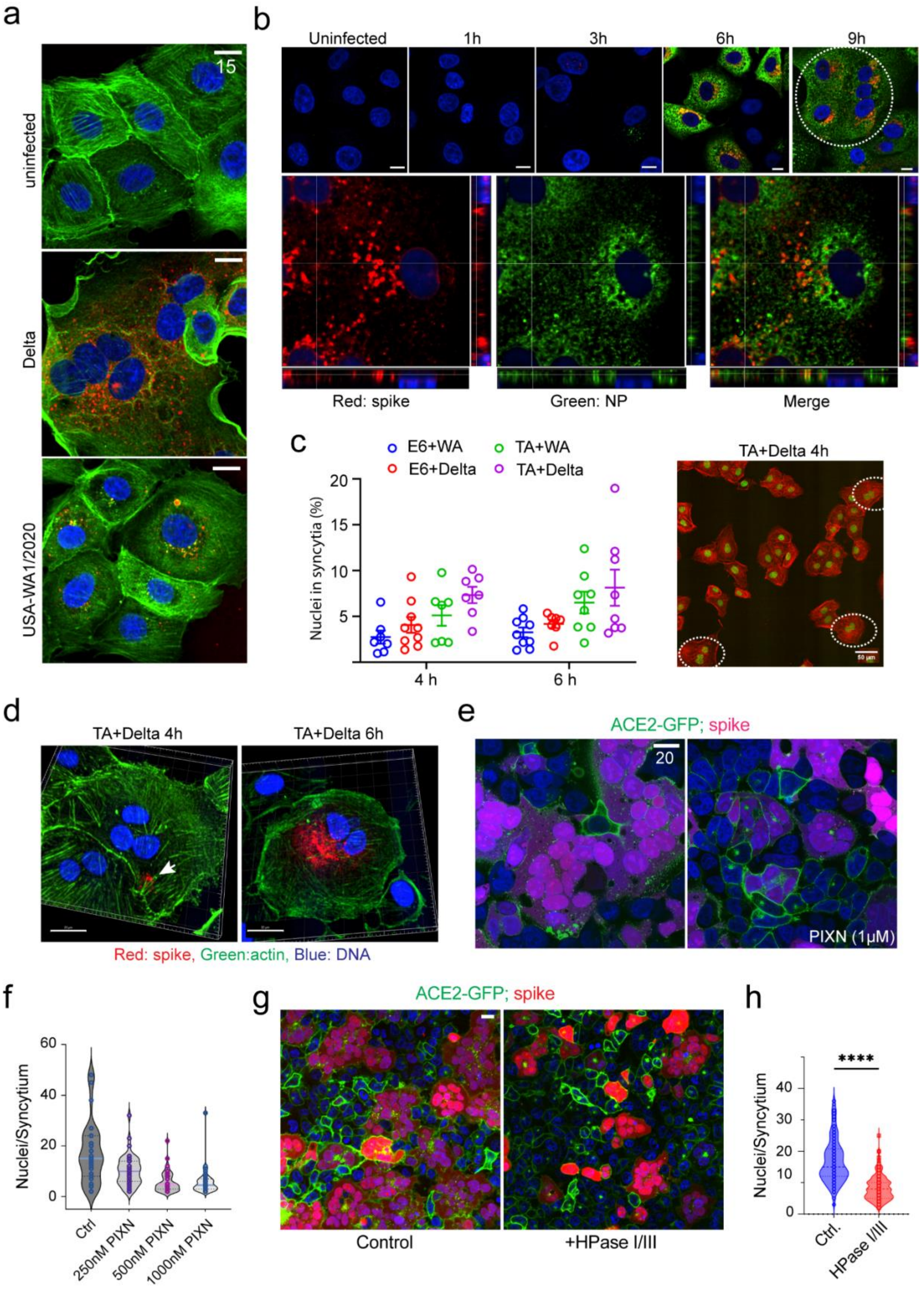
a
uninfected
Delta
USA-WA1/2020
15
b
Uninfected
1h
3h
6h
9h
Red: spike
Green: NP
Merge
c
E6+WA
E6+Delta
TA+WA
TA+Delta
Nuclei in syncytia (%)
4 h
6 h
TA+Delta 4h
d
TA+Delta 4h
TA+Delta 6h
Red: spike, Green:actin, Blue: DNA
e
ACE2-GFP; spike
20
PIXN (1μM)
f
Nuclei/Syncytium
Ctrl
250nM PIXN
500nM PIXN
1000nM PIXN
g
ACE2-GFP; spike
Control
+HPase I/III
h
****
Nuclei/Syncytium
Ctrl.
HPase I/III

Supplementary Fig. 3: The cell surface heparan sulfate promotes spike-induced cell-cell fusion.

a Vero TA6 cells were infected with the indicated SARS-CoV-2 variants (MOI=0.5) for 4 h and stained with anti-nucleocapsid protein (red) and the actin staining dye Alexa Fluor 488 Phalloidin (green). Scale bars, 15 μm. The images are representative of two biological repeats.

b Vero TA6 cells were infected with USA-WA1/2020 (MOI=0.5) for the indicated time periods (top panels). Cells were stained with Hoechst (blue), spike (red), and NP (green) to reveal syncytia, which were seen in cells 9 hpi (dashed circle). The bottom panels show enlarged view of a representative cell at 6 hpi, showing the ER and post-ER localization of de novo synthesized spike. Scale bars, 10 μm. The images are representative of two biological repeats.

c Vero E6 (E6) and Vero TA6 (TA) cells were infected with either the USA-WA1/2020 (WA) or Delta SARS-CoV-2 variant at a MOI of 0.01 for 4 or 6 hours. Cells were stained with Hoechst and an actin dye. The number of nuclei in syncytia was counted. The picture shows an example of small syncytia observed in TA cells infected with the Delta strain (dashed circles).

d 3D view of TA cells infected with the Delta strain at a MOI of 0.01 for the indicated time period. Note that de novo synthesized spike (ERGIC-localized) was not detected 4 h post infection.

e, f PIXN inhibits spike-induced cell-cell fusion. ACE2-GFP cells (green) were incubated with spike/mCherry-expressing cells (magenta) in the absence (Control) or presence of PIXN for 60 minutes. The graph in **f** shows the size of syncytia, as indicated by the number of nuclei per syncytium. Scale bars, 20 μm.

g, h Heparinase I/III treatment inhibits spike-mediated cell-cell fusion. ACE2-GFP cells (green) were either mock-treated or treated with Heparinase I/III (HPase I/III) for 3 h before incubation

with spike/mCherry-expressing cells (red). Scale bar, 10 μm. The graph in **h** quantifies the size of syncytia. ****, p<0.0001 by two-sided unpaired student's t-test, n=3 biological repeats.

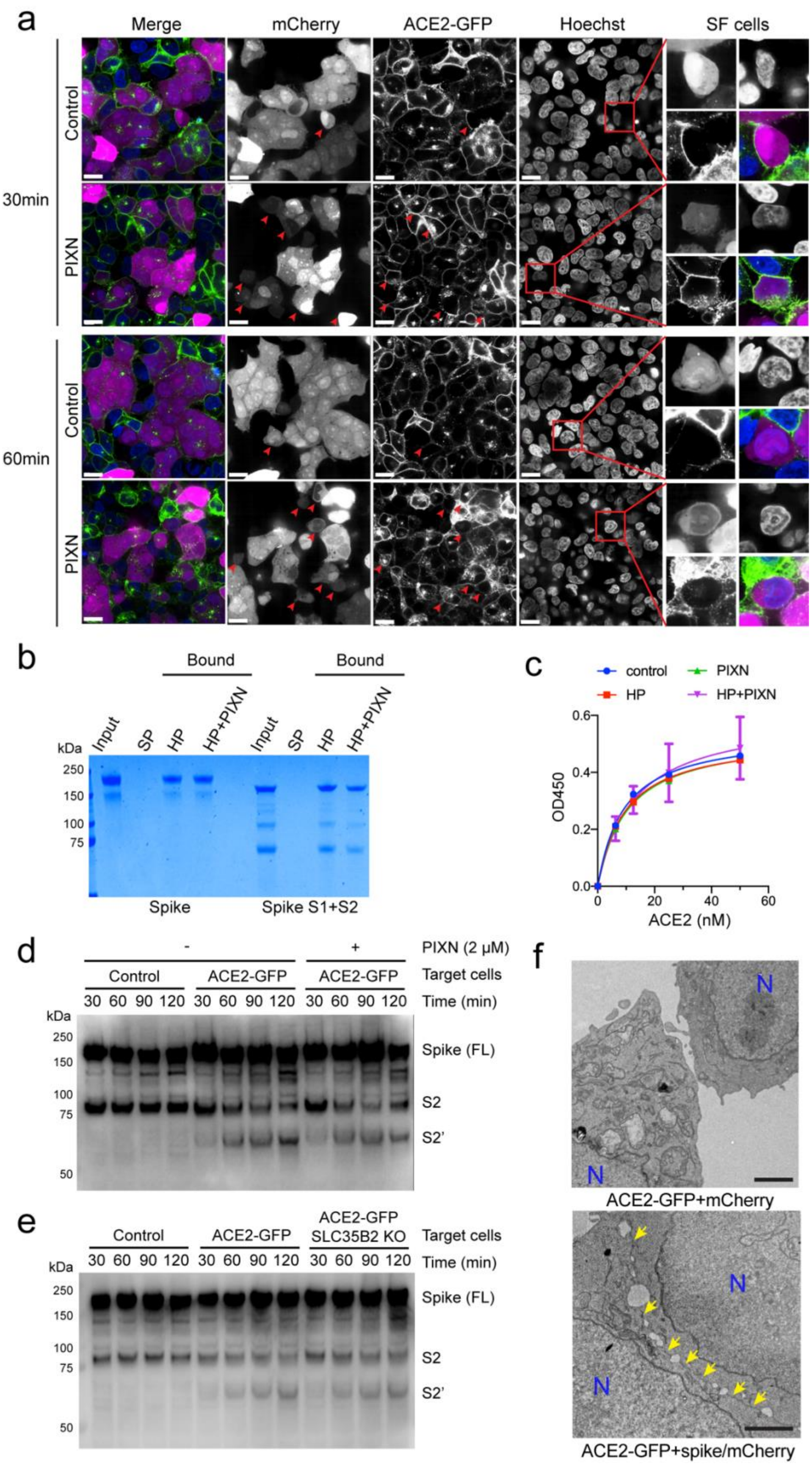
a
Merge
mCherry
ACE2-GFP
Hoechst
SF cells
30min
Control
PIXN
60min
Control
PIXN
b
Bound
Bound
kDa
Input
SP
HP
HP+PIXN
Input
SP
HP
HP+PIXN
250
150
100
75
Spike
Spike S1+S2
c
control
PIXN
HP
HP+PIXN
OD450
ACE2 (nM)
d
PIXN (2 μM)
-
+
Control
ACE2-GFP
ACE2-GFP
Target cells
30 60 90 120 30 60 90 120 30 60 90 120
Time (min)
kDa
250
150
100
75
50
Spike (FL)
S2
S2'
e
Control
ACE2-GFP
ACE2-GFP SLC35B2 KO
Target cells
30 60 90 120 30 60 90 120 30 60 90 120
Time (min)
kDa
250
150
100
75
50
Spike (FL)
S2
S2'
f
N
N
ACE2-GFP+mCherry
N
N
ACE2-GFP+spike/mCherry

Supplementary Fig. 4: PIXN inhibits spike-induced cell-cell fusion in a semi-fusion state.

a ACE2-GFP cells (green) were incubated with spike/mCherry-expressing cells (magenta) in the absence (Control) or presence of PIXN (2 μM) for 30 or 60 minutes. Shown are representative pictures with examples of semi-fusion (SF) cells highlighted (red boxes). Red arrows show more examples of SF cells. Scale bars, 20 μm. Images are representative of two biological repeats.

b PIXN does not inhibit the interaction of HS with the spike. The spike proteins (lanes 1-4, a mutant with the furin-cleavage site mutated; lanes 5-8, WT spike with a fraction cleaved by the furin protease) were incubated with Sepharose beads (SP), heparin-conjugated beads (HP), or heparin beads prebound with PIXN (HP+PIXN). The bound proteins and a fraction of the input (15%) were analyzed by SDS-PAGE followed by Coomassie blue staining. The gel is representative of two biological repeats.

c PIXN does not affect the spike-ACE2 interactions. The interaction of ACE2 with spike was measured by an ELISA-based plate assay with the indicated compounds present. HP, heparin. Error bars indicate means ± SD (n=3 biological repeats).

d, e PIXN does not inhibit ACE2-dependent proteolytic processing of the spike. ACE2-GFP cells or control cells without ACE2 overexpression (-) were incubated with spike/mCherry-expressing cells in the absence (Control) or presence of PIXN (2 μM) for the indicated time points. Cell extracts were analyzed by immunoblotting with an antibody detecting both fully length spike and the cleaved S2 and S2' fragment. Note that the conversion of S to S2' fragment, which depends on ACE2, is not affected by PIXN. The gels are representative of two biological repeats.

f The spike dependent formation of synapse-like cell-cell contracts. ACE2-GFP cells were mixed with mCherry transfected cells (Left) or spike/mCherry co-transfected cells (Right) for 12 minutes before fixing and analyzed by transmission electron microscopy. N, nucleus. The arrows indicate synapse-like cell-cell contacts formed between ACE2-GFP and Spike cells. Scale bar, 2 μm. The images are representative of two biological repeats.

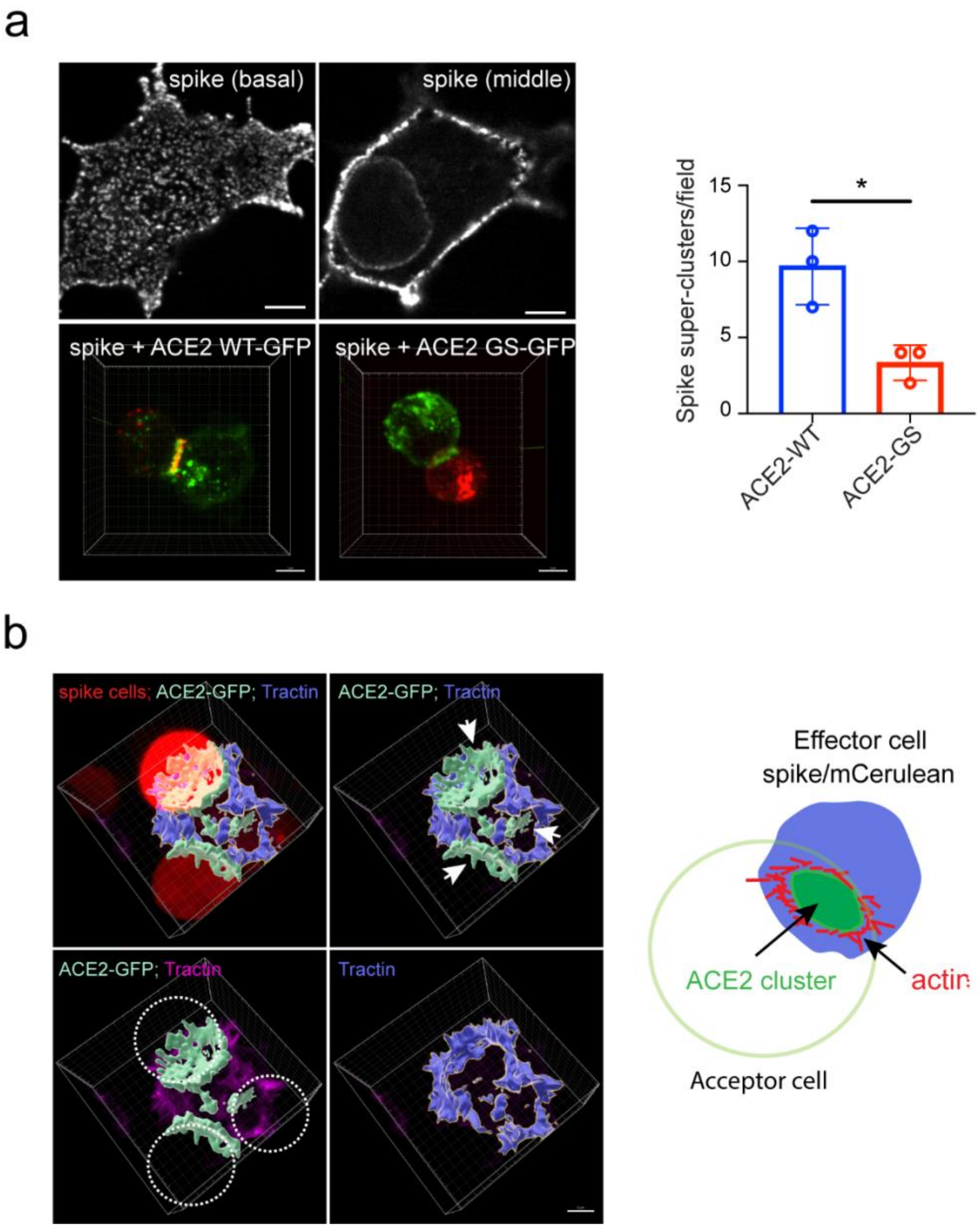

Supplementary Fig. 5: Heparan sulfate promotes the assembly of ACE2 super-clusters.

a WT ACE2 but not the ACE2 GS mutant induces spike super-cluster formation. Spike-transfected cells were incubated with medium (Top panels), ACE2 WT-GFP cells (Bottom left) or ACE2 GS-GFP cells (Bottom right) for 10 min. Cells were fixed and stained with spike antibodies (red). The top panels show two confocal sections while the bottom panels show a 3D view of the cells. Scale bars, 5 μm. The graph shows the number of spike super-clusters per fields. Error bars indicate means ± SD, n=3 biological repeats. *, p=0.02 by two-sided unpaired student's t-test.

b Synapse-like membrane contact sites containing ACE2 clusters are enriched in actin filaments. ACE2-GFP cells were transfected with mCherry-Tractin to label actin filaments and then co-cultured with spike/CFP co-transfected cells (red) for 5 minutes before 3D imaging. Arrows indicate 3 ACE2 clusters in surface-rendered 3D view (green). Note that F-Tractin-labeled actin filaments are concentrated around the ACE2 clusters. The dashed lines indicate CFP-positive effector cells. Scale bar, 5 μm. The diagram on the right is a schematic illustration of a spike-induced membrane synapse.

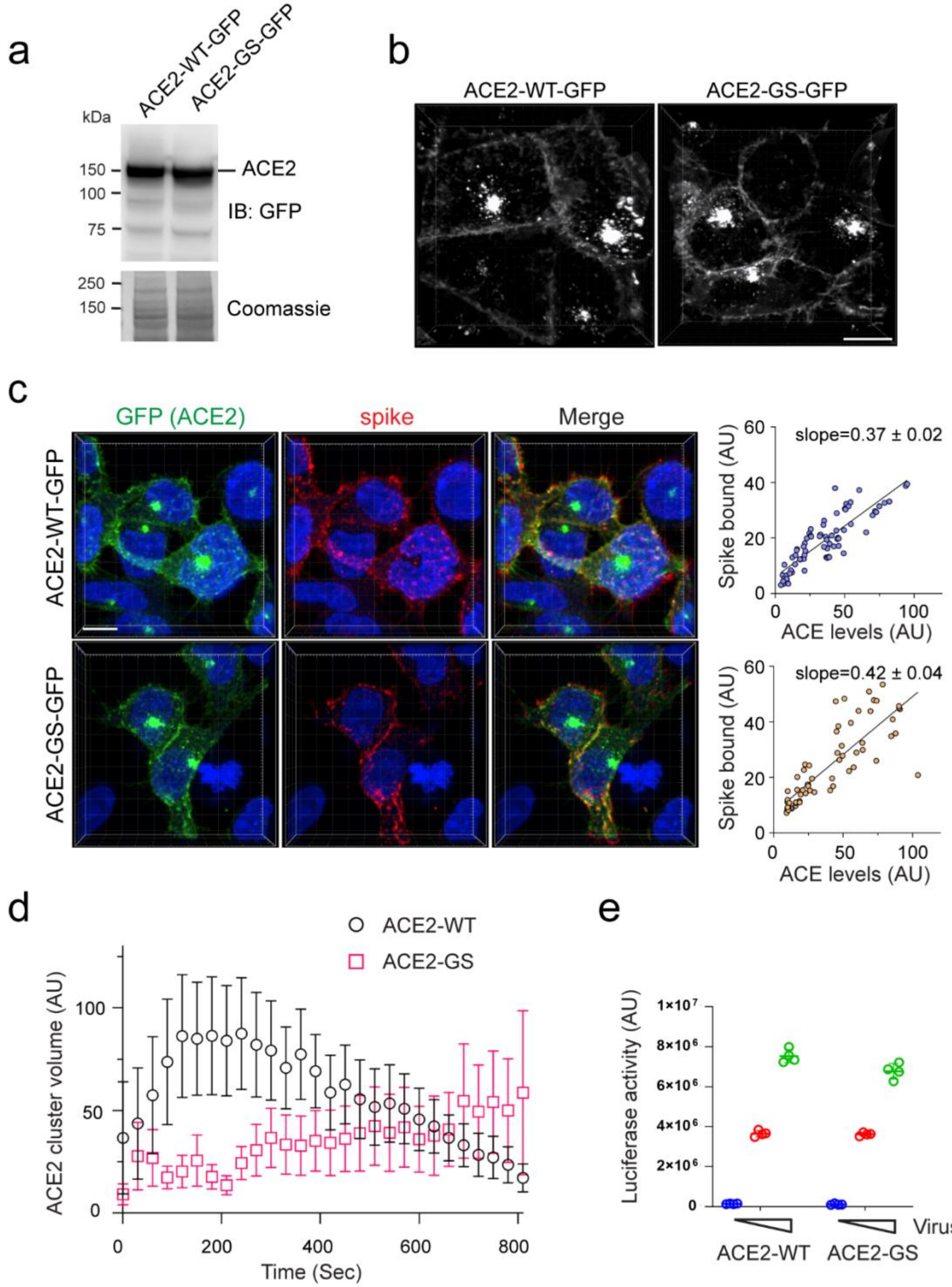

Supplementary Fig. 6: A linker segment in ACE2 facilitates spike-induced ACE2 clustering.

a ACE2-GS-GFP is expressed at a similar level as WT ACE2. Cell extracts of the indicated cell lines were analyzed by immunoblotting. The gel is representative of two biological repeats.

b ACE2-GS-GFP and WT ACE2-GFP are both localized to the plasma membrane and endocytic vesicles. Scale bar, 10 μm. The images are representative of three biological repeats.

c ACE2-GS-GFP and WT ACE2-GFP bind to the spike with a similar affinity. The indicated cell lines were incubated with the spike and stained with spike antibodies (red) and Hoechst (blue). The graphs show the amount of cell surface-bound spike, which correlates with ACE2 levels as measured by Image J.

d ACE2-GS-GFP cells are defective in forming ACE2 clusters when co-cultured with spike/mCherry cells. The graph shows the averaged 3D volume of ACE2 clusters from randomly selected live cell imaging videos. Error bars indicate means ± s.e.m., n=5 cells.

e The ACE2-GS mutant can mediate endocytosis-dependent entry of spike-coated pseudovirus. ACE2 WT-GFP or ACE2 GS-GFP cells were infected with increased dose of pseudovirus coated with the spike of the Washington strain. The efficiency of the viral entry, as determined by the expression of the luciferase was plotted.

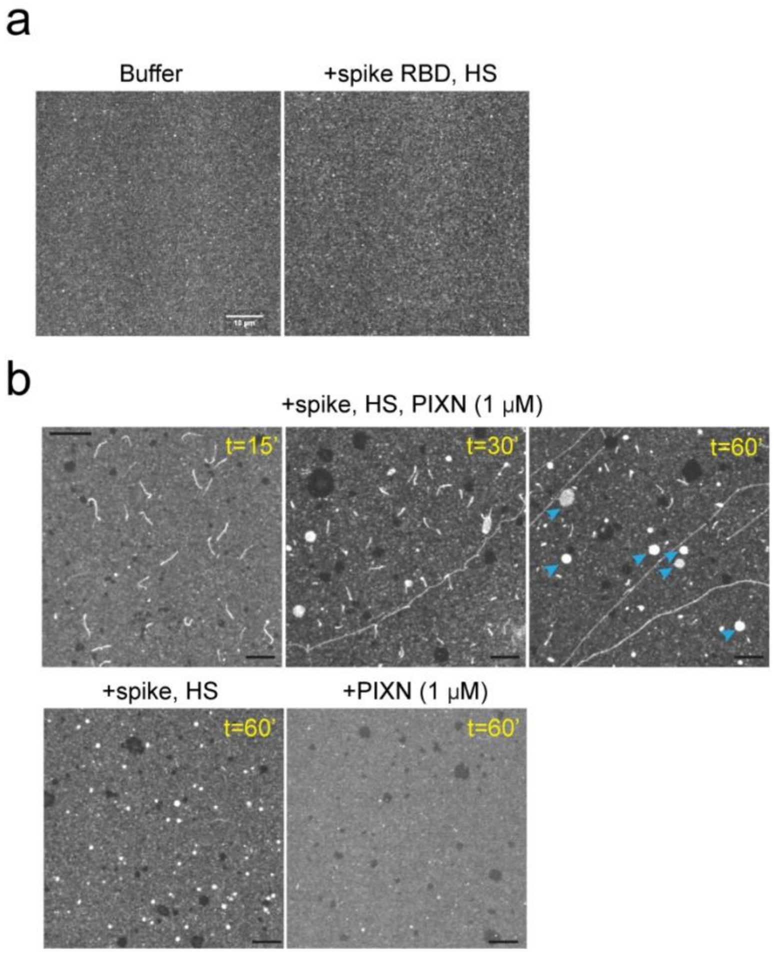

Supplementary Fig 7: PIXN alters the morphology of ACE2 clusters in the presence of spike and HS.

a Fluorescence-labeled ACE2 $_{1\text{-}740}$ was anchored to lipid bilayers on a cover glass and treated as indicated for 60 min. Scale bar, 10 µm. The images are representative of two biological repeats.

b Fluorescence-labeled ACE2 $_{1\text{-}740}$ was anchored to lipid bilayers on a cover glass and treated as indicated. Note that PIXN alters the shape of clustered ACE2, forming filament-like structures instead. Additionally, some round ACE2 clusters were observed (blue arrowheads)

but they are much bigger than those formed in the absence of the drug. Scale bar, 10 µm. The images are representative of three biological repeats.

Supplementary table 1: HS assignments and calculated Δppm for the NMR experiment.

	6merNS2S3S6S		6merNS2S3S6S+PXT			
	^{1}H Chemical Shift (ppm)	^{13}C Chemical Shift (ppm)	^{1}H Chemical Shift (ppm)	^{13}C Chemical Shift (ppm)	1H Δppm	13C Δppm
H1-A	5,383	102.12	5,357	101.96	0.026	0.16
H2-A	3,810	75.19	3,809	75.19	0.001	0
H3-A	4,056	78.52	4,058	78.53	0.002	0.01
H4-A	4,003	80.48	4,016	80.25	0.013	0.23
H5-A	4,110	79.72	4,111	79.7	0.001	0.02
H1-B	5,669	100.53	5,682	100.46	0.013	0.07
H2-B	3,404	60.87	3,406	60.95	0.002	0.08
H3-B	3,751	72.44	3,819	71.83	0.068	0.61
H4-B	3,892	78.34	3,918	77.81	0.026	0.53
H5-B	4,053	72.10	4,073	72.43	0.02	0.33
H6a-B	4,555	69.05	4,554	69.12	0.001	0.07
H6b-B	4,318	69.05	4,323	69.12	0.005	0.07
H1-C	5,250	102.41	5,311	101.48	0.061	0.93
H2-C	4,406	80.62	4,429	80.41	0.023	0.21
H3-C	4,243	74.05	4,270	73.57	0.027	0.48
H4-C	4,249	79.09	4,271	78.79	0.022	0.3
H5-C	4,834	73.46	4,892	73.31	0.058	0.15
H1-D	5,634	98.77	5,656	98.6	0.022	0.17
H2-D	3,528	59.48	3,555	59.67	0.027	0.19
H3-D	4,443	79.01	4,462	78.97	0.019	0.04
H4-D	4,059	75.58	4,073	76.05	0.014	0.47
H5-D	4,261	72.36	4,243	72.48	0.018	0.12
H6a-D	4,565	68.84	4,575	68.81	0.01	0.03
H6b-D	4,356	68.84	4,369	68.81	0.013	0.03
H1-E	4,705	103.87	4,723	104.14	0.018	0.27
H2-E	3,504	75.61	3,505	75.6	0.001	0.01
H3-E	3,932	79.77	3,927	79.72	0.005	0.05

H4-E	3,925	79.19	3,923	78.86	0.002	0.33
H5-E	3,852	79.94	3,893	79.79	0.041	0.15
H1-F	5,703	100.35	5,689	100.37	0.014	0.02
H2-F	3,333	60.83	3,334	60.84	0.001	0.01
H3-F	3,718	74.01	3,717	74.01	0.001	0
H4-F	3,669	71.87	3,664	71.86	0.005	0.01
H5-F	3,978	72.54	3,975	72.56	0.003	0.02
H6a-F	4,465	69.10	4,464	69.11	0.001	0.01
H6b-F	4,237	69.10	4,241	69.11	0.004	0.01

Supplementary table 2: A list of key reagents used in the study.

REAGENT	SOURCE	Catalog Number
**Antibodies, recombinant proteins, and dyes**		
Mouse Anti-Heparan Sulfate	US Biological	H1890
Rabbit Anti-SARS-Cov/SARS-Cov-2 Nucleocapsid	Sino Biological	40143-R001
Rabbit Anti-SARS-Cov-2 spike	Sino Biological	40592-R0004
Mouse Anti-SARS-Cov-2 spike	GeneTex	GTX632604
CSPα/DNAJC5	Lsbio	LS-C22593
Purified hACE2 extracellular domain	Sino Biological	10108-H08H-B
Alexa Fluor™ 488 Phalloidin	ThermoFisher	A12379
Hoechst 33,342, Trihydrochloride, Trihydrate	Thermo Fisher	H3570
**Chemicals, lipids, enzymes, and biological kits**		
Hellmanex III	Sigma	Z805939
Pixantrone	Skelleckchem	S5059
Mitoxantrone	Skelleckchem	S2485
Synthesized heparan sulfate 6-mers	UNC at Chapel hill	In house
Heparin sodum salt	Sigma	H3149
Heparin sulfate	Sigma	H7640
LC1519	Univeristy of Minnesota	In house
LC1520	Univeristy of Minnesota	In house
LC1521	Univeristy of Minnesota	In house
LC1539	Univeristy of Minnesota	In house
LC1540	Univeristy of Minnesota	In house

LC1541	Univeristy of Minnesota	In house
LC1542	Univeristy of Minnesota	In house
LC1553	Univeristy of Minnesota	In house
POPC	Avanti	850457C
DOGS-NTA	Avanti	790404C
PEG-5000 PE	Avanti	880230C
Proteinase K	Thermofisher Scientific	AM2546
Heparinase I	NEB	P0735S
Heparinase III	NEB	P0737L
RNeasy Plus Mini Kit	Qiagen	74136
QuantiNova SYBR Green PCR kit	Qiagen	208052
2019-nCoV RUO Kit	IDT	10006713
cDNA Reverse Transcription Kit	Thermofisher Scientific	4368813
Bright-Glo Luciferase kit	Promega	E2620
CellTiter-Glo cell viability kit	Promega	G7572
**Plasmids and viruses**		
pCMV-hACE2-GS-GFP	Genewiz	Contract Service
pLV-Mcherry	Addgene	36084
pCMV-SARS-CoV-2-Spike (Delta)	Codexbiosolutions	Contract Service
SARS-CoV-2 pseudoviral particles	Codexbiosolutions	Contract Service
SARS-CoV-2 isolate USA-WA1/2020 (Washington)	BEI Resources	NR-52281
SARS-CoV-2 isolate USA/MD-HP05647/2021(Delta)	BEI Resources	NR-55672
**Cell lines and mice**		
293T ACE2-WT-GFP stable clone	NIH	In house by Codex
293T ACE2-GS-GFP stable clone	NIH	In house by Codex
Vero E6	ATCC	CRL-1586
Vero E6-TMPRSS2 (TE6)	BPS Bioscience	78081
Vero E6-TMPRSS2-T2A-ACE2 (TA6)	BEI Resources	NR-54970
B6.Cg-Tg(K18-ACE2)2Prlmn/J (K18-hACE2) mice	JAX	34680
Calu-3	ATCC	HTB-55
3D EpiAirway model	Mattek	Custom service
