# Supplementary material for: Host heparan sulfate promotes ACE2 super-cluster assembly and enhances SARS-CoV-2-associated syncytium formation
Source: Nat Commun. 2023 Sep 18;14:5777. doi: 10.1038/s41467-023-41453-w (PMC10507024; doi:10.1038/s41467-023-41453-w)
Supplement: Supplementary file 3 — Description of Additional Supplementary files [file 41467_2023_41453_MOESM3_ESM.pdf]

Description of additional supplementary files

File name: Supplementary Movie 1
Description: A 3D view of Vero TA6 cells infected with the Delta strain. A rotational view of Vero TA6 cells infected at a MOI of 0.01 for 4 h. Cells were stained with antibodies against spike (red), an actin dye (green), and Hoechst (blue). Note the spike signals (red) are localized on the surface of the infected cells.

File name: Supplementary Movie 2
Description: 4D live imaging of spike-induced cell-cell fusion. A spike/mCherry-expressing cell (magenta) contacts an ACE2-GFP cell (green), which induced the clustering of ACE2 at the cell-cell contact site. As the cluster grows, mCherry is leaked into ACE2-GFP cells, which is followed by the formation of a visible pore. The pore eventually expands to cause the complete fusion of the two cells.
